# Partial substitution of anthracite for coke breeze in iron ore sintering

**DOI:** 10.1038/s41598-021-80992-4

**Published:** 2021-01-15

**Authors:** Xin Zhang, Qiang Zhong, Chen Liu, Mingjun Rao, Zhiwei Peng, Guanghui Li, Tao Jiang

**Affiliations:** School of Minerals Processing and Bioengineering, Central South University, Changsha, 410083 People’s Republic of China

**Keywords:** Chemical engineering, Fossil fuels

## Abstract

In the sintering of iron ores, the partial substitution of anthracite for coke breeze has been considered to be an effective way of reducing pollutant emissions and production cost. In this study, the basic characteristics of anthracite and coke breeze were compared and the sintering performance at different substitution ratios of anthracite for coke breeze was investigated. The porosity of anthracite is lower than that of coke breeze, but its density and combustion reactivity are higher. The substitution of anthracite for coke breeze has no influence on the granulation effect of the sintering blend. As the anthracite proportion increased, the sintering speed and productivity increased and the sintering yield and tumbler index decreased. As the substitution ratio increased from 0 to 60%, the melting temperature duration and the melt quantity index decreased from 2.59 to 2.03 min and from 3218.28 to 2405.75 °C·min, respectively, leading to insufficient sintering mineralization and bad sintering indexes. For an anthracite substitution ratio of 40%, the sintering speed, sintering productivity, sintering yield and tumbler index were 22.34 mm min^−1^, 1.49 t·(m^2^ h)^−1^, 71.65% and 63.59%, respectively, which entirely satisfies the production requirements. Furthermore, hematite (Fe_2_O_3_), calcium ferrite (CaO–Fe_2_O_3_), and compound calcium ferrite (CaO–SiO_2_–Fe_2_O_3_) were the major mineral phases, which were embedded with an interwoven structure.

The production of crude steel in the iron and steel industry in China has grown dramatically, which has made the country the world's largest steel producer since 1996^1–3^. 40%–60% of the total iron-containing burden in the blast furnace during ironmaking is sinter^4^. The iron ore sintering process is one of the vital steps -in an integrated steelmaking chain, the goal of which is to agglomerate iron ore fines into porous sinters with a high mechanical strength and high thermal and reducing behaviors. This process is characterized by energy and pollution intensity, accounting for about 9–12% of the total energy used in the iron and steel industries^5–7^.

During the sintering process, sinter fuels are commonly used to provide the required energy. The coke breeze comes from coking plant (typically less than 3 mm in size) and is widely used due to its unique combustion properties, and its mechanical and structural properties. Fuel costs are high for sintering plants because of the expensive price of coke. Moreover, coke breeze causes more environment pollution because of higher NO_x_ emission than that of anthracite. The contradiction between the coke supply and demand, and the environmental problems caused by the coke fines lead to the high price^8,9^.

To address this problem, researchers are beginning to search for other fuels for completely or partially substituting coke breeze. Biomass is a renewable and sustainable energy source, and it has now become an increasing important alternative energy source in the iron ore sintering process^10,11^. Unlike traditional fuels, biomass is an environmentally friendly resource that is carbon neutral and has lower CO_2_, NO_X_, and SO_2_ emissions, making it beneficial for alleviating environment-protection pressure. Biomass fuel also differs from conventional fuels with respect to its density, porosity, reactivity, and combustibility^12^. However, the different biomass fuels used in the sintering process cause issues such as volatile matter pyrolysis of the uncarbonized biomass. This leads to poor fuel utilization, and a higher chemical activity of the charcoal, which damages the sinter quality when a higher proportion of coke breeze is replaced with biomass. Thus, several measures were researched, for example, adding more reactive iron ores, using coarse charcoal, and adjusting the granulation methods to coat the charcoal particles and form granules^10,13,14^. However, these measures increase operating costs. Moreover, hydrogen-based gaseous fuel has used as another substitution fuel. It can reduce the CO_2_ emission and improve energy efficiency, but need extra energy efficiency, which also increases the cost in iron ore sintering^15,16^. Semi-coke, BF dust and sludge have also used as partial substituting fuels, however, when at a high semi-coke proportion problems like fuel combustion efficiency, an increase of SO_x_ and zinc emission for BF dust and sludge substitution enmerges^17,18^.

Based on the above issues, anthracite has become the main substituting fuel for coke breeze in iron ore sintering due to its reasonable price^19,20^. When this substitution is made, the particle size of the anthracite needs to be controlled^21^. The utilization of anthracite increases the thickness of the combustion zone, which influences the quantity and quality of the finished sinter. According to a basic performance study, the reactivity of anthracite is lower than that of coke breeze, which is attributed to the differences in their true surface areas and intrinsic surface reactivities. Through mathematical modeling simulations, the effect of anthracite on iron ore sintering was evaluated, and it was determined that its combustion characteristics are not as good as those of coke breeze^19,22^. However, the combustion performances of various anthracite differ.

This study focused on determining the basic physical and combustion characteristics of anthracite and the differences between the characteristics of anthracite and those of coke breeze. The combustion characteristics of both were researched by through thermogravimetric analysis (TGA), which covered a wide range of applications in the research, development, and economic assessment of coal and other fuels^23–25^. Experiments involving the substitution of anthracite for coke breeze were conducted to assess the effects on the sintering process, including the granule size distribution, sintering indexes, and thermal bed characteristics, and to study the influences on the mineral composition and microstructure of the sinter.

## Experimental materials and methods

### Raw materials

In this study, the raw materials consisted of iron-bearing materials (iron concentrates, iron ore fines, and miscellaneous materials), fluxes (limestone, dolomite, quicklime, and serpentine), solid fuels (coke breeze and anthracite), and return fines. The chemical compositions and size distributions of all of the raw materials are presented in Tables 1 and 2. The iron-bearing materials regulated the Fe and SiO_2_ contents, and the fluxes adjusted the sinter basicity (CaO/SiO_2_, mass ratio) and MgO content. The basicity of the finished sinter was maintained at 1.70. As can be seen from Table 2, the average particle size of the anthracite was 1.97 mm, which is a little larger than that of the coke breeze. Table 3 shows the results of the proximate analysis of the coke breeze and anthracite. The anthracite contained 7.73% volatiles, and it contained more fixed carbon and had a lower ash content than the coke breeze. It can be seen that the contents of N and S in anthracite are obviously lower than that of coke breeze, indicating that anthracite is more environmentally friendly in sintering process. Moreover, the heating value of the anthracite was higher than that of the coke breeze.

**Table 1:** Chemical compositions of all of the raw materials in the sinter mixture (mass %).

Raw materials	TFe	CaO	SiO_2_	MgO	Al_2_O_3_	FeO	LOI	Proportion
Iron concentrates	65.31	0.26	4.04	0.07	1.24	0.28	0.45	59.31
Iron ore fines	43.80	0.13	11.83	0.25	1.58	2.55	6.70	2.37
Miscellaneous materials	43.80	7.15	5.48	1.01	1.53	19.27	21.30	2.23
Return fines	55.27	8.01	6.88	1.01	1.65	5.61	1.05	20.27
Limestone	0.78	50.85	4.05	0.42	0.76		40.42	2.85
Dolomite	2.33	26.81	1.50	19.31	0.45		44.11	2.47
Quicklime	0.27	90.15	2.13	0.45	0.49		3.64	5.00
Serpentine	5.54	2.37	39.65	32.47	1.80	5.27	11.93	1.00
Coke breeze	2.72	1.29	10.22	0.35	5.15		77.52	–
Anthracite	1.21	0.61	8.57	0.15	3.65		84.16	–

*LOI* loss on ignition.

**Table 2:** Size distributions of all of the raw materials (mass%).

Size (mm)	+ 8	8–5	5–3	3–0.9	0.9–0.5	0.5–0.1	0.1–0.074	Average size
Iron concentrates	4.36	4.96	5.66	5.61	2.49	50.93	26.00	1.18
Manganese ore fines	10.75	7.91	12.44	15.84	8.55	24.7	19.81	2.32
Miscellaneous materials	1.24	2.05	7.35	12.28	6.85	38.81	31.42	0.96
Return fines	0.07	2.71	20.00	32.23	12.59	25.4	7.01	1.77
Limestone	1.65	9.88	18.11	21.03	7.59	21.9	19.84	2.03
Dolomite	1.27	2.55	32.94	41.17	9.95	4.94	7.17	2.48
Serpentine	0.00	0.72	14.62	34.54	17.03	26.75	6.34	1.49
Coke breeze	0.26	3.15	18.17	24.05	14.29	31.83	8.24	1.60
Anthracite	0.36	3.91	22.48	32.42	14.43	18.85	7.56	1.97

**Table 3:** Proximate analysis, ultimate analysis, and heating values of the fuels (mass%, MJ kg^−1^).

Fuel types	Proximate analysis			Ultimate analysis					Qnet.d (MJ kg^−1^)
	Fixed carbon	Volatiles	Ash	C	H	O	N	S	
Coke breeze	73.11	4.41	22.48	74.36	2.52	8.86	1.03	0.26	31.89
Anthracite	76.46	7.73	15.84	77.63	2.70	7.91	0.19	0.19	32.45

## Methods

### Thermogravimetric analysis

A NETZSCH STA-449C differential thermogravimetric analyzer (precision of temperature measurement ± 1 °C, microbalance sensitivity of 0.1 μg) was used for combustion tests of the coke breeze and anthracite. The weight-loss values and the rates of the fuels as functions of temperature or time were recorded continuously under dynamic conditions. Precisely Weighed 10 ± 0.1 mg samples (particle size of 0.125–0.25 mm) were thinly distributed in an Al_2_O_3_ ceramic crucible, which eliminated the effects of the inevitable side reactions and the mass and heat transfer limitations. In the combustion tests on coke breeze and anthracite, the heating temperature ranges from 20 to 1000 °C and the heating rate and flow rate of the artificial air were fixed at 10 °C min^−1^ and 100 ml min^−1^, respectively.

The typical parameters generally include the ignition temperature (T_i_), the peak temperature of the maximum weight loss rate (T_m_), the burnout temperature (T_h_), the maximum combustion rate (DTG_max_), the mean combustion rate (DTG_mean_) and the burnout time (τ). On the combustion profile, the ignition temperature refers to the start of a sudden decrease in fuel weight; the burnout temperature represents the point at which combustion is complete; and the τ is the time needed for the coke breeze and anthracite combustion to progress from ignition to burnout. The DTG_max_ is when the weight loss rate reaches the maximum; and the DTG_mean_ is the mean rate of weight loss. In addition, the overlapping combustion interval of the fuel is the weight loss of the anthracite divided by the total weight loss of the coke breeze in the combustion interval^23–25^.

### Sintering pot trials

**Figure 1:**
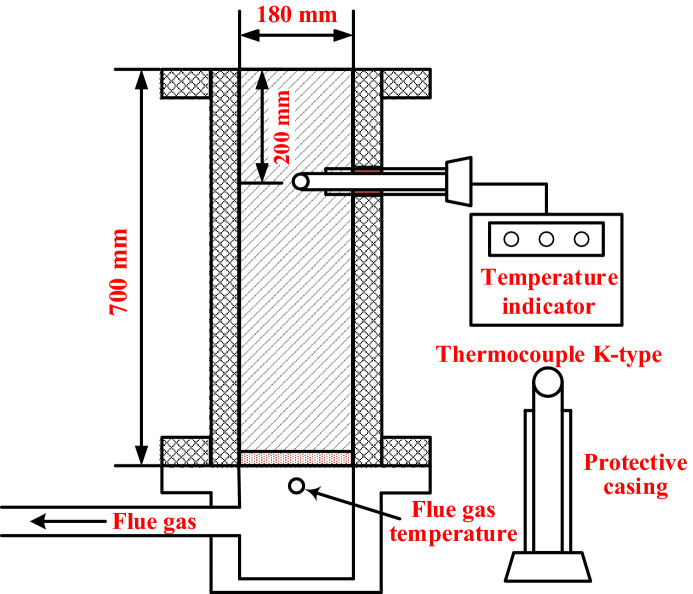
Schematic of the sinter pot used in the sintering experiments.

The sintering experiments were conducted in a laboratory-scale sinter pot with a depth of 700 mm and a diameter of 180 mm. A schematic of the experiment is shown in Fig. 1^6,26,27^. The raw materials were blended manually according to the proportions presented in Table 1. During this stage, the sinter mixture’s moisture was controlled at 7.1 ± 0.1% to meet the prescribed level. Then, the sinter mixture was granulated into sinter feed in an electric drum granulator with length a of 1400 mm and a diameter of 600 mm. The revolving speed and duration were 15 rpm and 5 min, respectively. A hearth layer was prepared at the bottom of the sinter pot to protect the grate from high-temperature erosion (1 kg sintering production, size of 10–15 mm). After the sinter feed was placed in the pot, an ignition hood fueled by natural gas was used to ignite the fuels and to supply enough heat. The ignition temperature, time, and negative pressure were the conventional values of 1050 ± 50 °C, 1 min, and 6 kPa, respectively. The pressure drop was immediately adjusted to 12 kPa after ignition and then to 6 kPa for cooling when the flue gas temperature reached the maximum value. Then, the combustion front could move downward, and it was sustained by an induced draft fan. A thermal couple (K-type) was installed 200 mm from the sinter bed’s surface to measure the temperature profile.

The proportions of anthracite substituted for coke breeze in the sinter pot experiments were 0%, 20%, 40%, and 60%, and the effects on the granule size distribution, the sintering indexes, and the thermal characteristics were investigated. The proportion of the sintering blending was set according to the basic values shown in Table 1, including the total solid fuel ratio of 4.5%. The sintering basicity and sinter mixture moisture were 1.7 and 7.1%, respectively.

### Evaluation of the sintering performance

The sintering speed, yield, tumbler index, and productivity are four vital indexes used to evaluate the sinter quality^28^. Sintering speed is the height of the sinter bed divided by the sintering time (mm min^−1^).The yield is the mass of the > 5 mm sinter divided by the total sinter mass minus the hearth layer mass of 1 kg (%). The productivity is the production capacity that the sintering machines can reach for a specific area and time (t m^−2^ h^−1^). The sintering speed, yield and productivity are calculated according to Eqs. (1)–(3):

1 $$SS = \frac{H}{t},{\text{mm}}\;{\text{mi}}{{\text{n}}^{ - 1}}$$

2 $$Yield = \frac{{M - {M_0}}}{M} \times 100,\%$$

3 $$P = 0.06 \times \left( {\frac{{M - {M_0}}}{S \times t}} \right),{\text{t}}\,{{\text{m}}^{ - 2}}\,{{\text{h}}^{ - 1}}$$

where H is the height of sintering bed (mm); t is the sintering time (min); M is the mass of sinter cake (kg); M_0_ is the mass of return fines (kg); S is the cross-sectional area of sinter bed (m^2^).

The tumbler index is the mass proportion of the > 6.3 mm sinter after 8 min tumbling, which is determined by ISO tumble standard. In addition, the return fines balance is the mass ratio of the produced return fines to the provided return fines; its reference value was within 1 ± 0.05. It was important that each sinter experiment reached the return fines balance, which guaranteed the validity of the four indexes.

### Quantitative parameters

**Figure 2:**
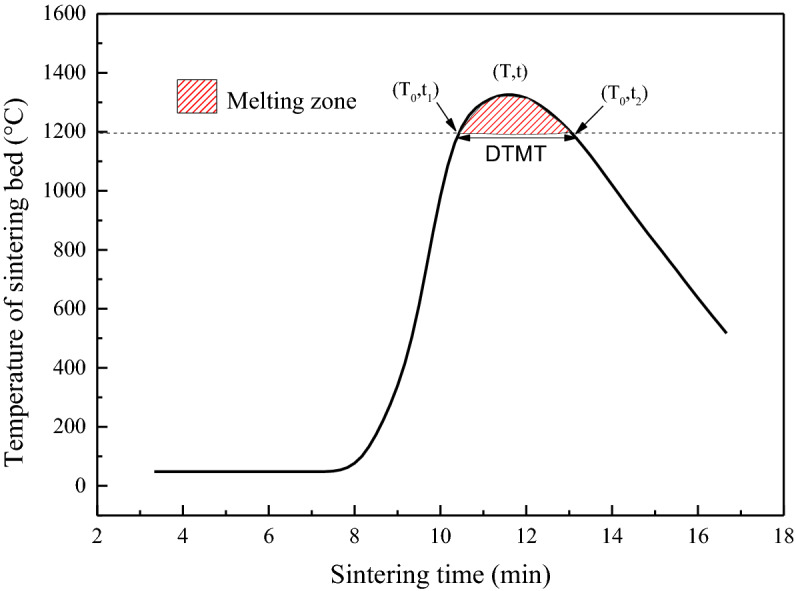
DTMT and melting zone of the sintering bed temperature profile.

It is known that the quality of sinter is highly related to the thermal status of the sintering bed. To quantitatively evaluate the thermal characteristics, the duration time of the melting temperature (DTMT) and the melt quantity index (MQI) were obtained from the temperature–time profile (Fig. 2).

The DTMT was defined as the time interval between the time at which the bed temperature reaches 1200 °C and the time at which the bed temperature decreases to below 1200 °C. The temperature of 1200 °C was selected as the base temperature of the melting zone, because calcium ferrite and compound calcium ferrite (SFCA) were the major bonding phases used to bond separate particles together, during the sintering process, so the melting point was slightly above 1200 °C. Therefore, a sufficient DTMT was a precondition to generate enough of the melting phase.

The MQI is defined as the area above 1200 °C on the sintering bed temperature–time profile. It is a comprehensive parameter reflects the melting zone information. A larger MQI value indicates that more melting phase is formed, thus contributing to sufficiently filling the voids between the separate particles and combining them together after cooling. It is expressed by Eq. (4):

4 $$MQI = \mathop \smallint \limits_{t_1}^{t_2} \left( {T - {T_0}} \right)dt$$

where T_0_ is the initiating temperature of 1200 °C; T is the bed temperature at time t (°C); t_1_ and t_2_ are the starting time and ending time of the 1200 °C (min) phase, respectively.

## Results

### Characteristics of anthracite and coke breeze

#### Density and porosity

**Figure 3:**
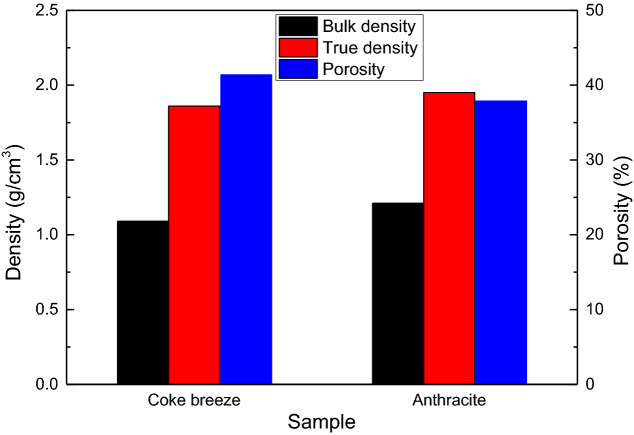
Densities and porosities of the coke breeze and anthracite.

Figure 3 shows the differences in the densities (bulk density, true density) and porosity of the coke breeze and anthracite used in the experiments. As is shown in the Fig. 3, the bulk density and true density of the anthracite were slightly higher than those of the coke breeze, that is, the volume of the anthracite added to sinter mixture was less than that of the coke breeze for the same mass. The porosity of the anthracite was 37.9%, which was lower than that of the coke breeze (41.4%). In general, the bulk densities, true densities and porosity of coke breeze and anthracitewere similar. These properties have no effect on the substitution anthracite for coke breeze in iron ore sintering.

#### Combustion characteristics

**Figure 4:**
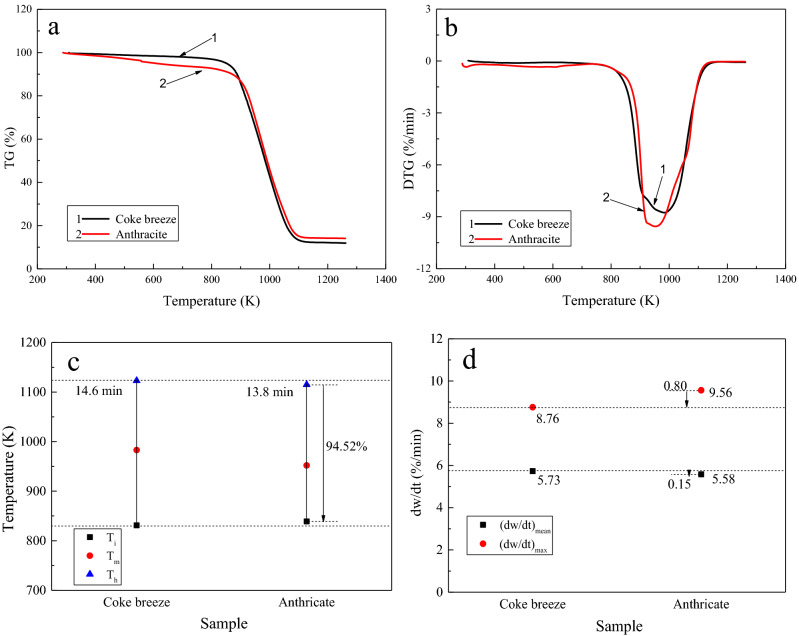
Combustion behaviors of coke breeze and anthracite (**a**) TG profile, (**b**) DTG profile, (**c**) combustion temperatures and burnout times, and (**d**) maximum weight loss rate and mean weight loss rate.

The TGA experimental conditions, the temperature, and the duration of the high temperature were far different from those of real sintering processes; however, these experiments are a simple but quite effective technique for observing the burning profile of fuels^29–31^. Therefore, the different combustion behaviors of coke breeze and anthracite during the sintering process can be estimated from the combustion characteristics determined from the TGA. Figure 4 shows the results of the combustion behaviors of the coke breeze and anthracite. As is shown in Fig. 4, b, both the anthracite and coke fines exhibit only one combustion peak since the combustion of the fixed carbon contributes greatly to their weight loss. Their basic variation trends during the temperature increase process are similar. As can be seen from Fig. 4c, d , the coke breeze had an ignition temperature of 558 °C and a burnout temperature of 850 °C, while those of anthracite were 566 °C and 842 °C, respectively. Both ignited at a similar temperature. The overlapping combustion interval between the coke breeze and anthracite was 94.52%, indicating that they have similar combustion behaviors. In addition, a meaningful difference in the details. First, anthracite burns completely, leading to a shorter combustion time of 13.8 min; while coke breeze burns out at a longer combustion time of 14.6 min. The DTG_max_ of anthracite is higher (0.80%/min) than that of coke breeze, indicating that it has a fiercer combustion, but its DTG_mean_ is slightly lower (0.15%/min). The combustion characteristics of these two fossil fuels make it clear that the combustion reactivity of anthracite is greater than that of coke breeze, which can affect the combustion conditions in the sintering bed.

### Assessment on the substitution of anthracite for coke breeze

#### Effect on granule size distribution

**Figure 5:**
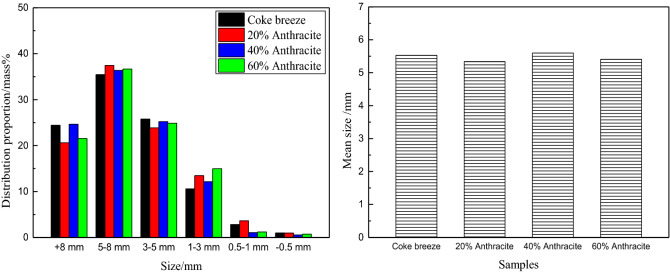
Effect of substituting anthracite for coke breeze on granule size distribution.

The effect of substituting anthracite for coke breeze on the granule size distribution is shown in Fig. 5. As can be seen, the granulating effect was good in all of the experiments^32^. The ratio of the + 3 mm sintering granules of was much greater than that of the − 3 mm sintering granules, and the 5–8 mm particles accounted for the largest percentage. In particular, the granule distribution proportions of + 8 mm, 5–8 mm and 3–5 mm particles were very similar in the coke breeze experiments and of the experiments in which anthracite was substituted for coke breeze. The mean size of the sinter mixture in each sintering experiment ranged from 5 to 5.5 mm, resulting in a similar granulating effect. In short, the results of the granulating analysis suggest that substituting different amounts of anthracite for coke breeze does not have a negative influence, when the initial sinter bed permeability is the same.

#### Effect on sintering quality

**Figure 6:**
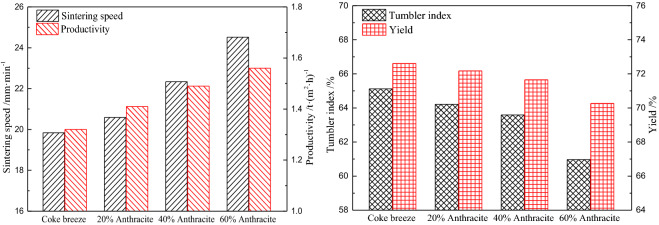
Effect of substituting anthracite for coke breeze on the sintering indexes.

The influence of substituting anthracite for coke breeze on sintering index is shown in Fig. 6. Compared with the base trial where only coke breeze was used, the sintering speed increased from 19.84 to 24.52 mm min^−1^ as the proportion of anthracite substitution increased. The improvement of the sintering speed was positively correlated with the shorter combustion time of the anthracite. The sinter yield worsened to a small extent, decreasing from 72.61 to 70.26%, which still meets the production requirement for the laboratory sintering experiment. Taking the sintering speed increase and the yield decrease into consideration, based on our calculations, substituting anthracite for coke breeze improves the productivity by 18.18%. However, the last important indicator, that is, the tumbler index, deteriorated significantly when the replacement percentage reached 60%. For a substitution proportion of 40%, the sinter tumbler index retained at 63.59%, which satisfies the quality requirements. To achieve ideal sintering indexes, the proportion of anthracite substituted for coke breeze should not exceed 40%.

#### Effect on the thermal characteristic of the sintering bed

**Figure 7:**
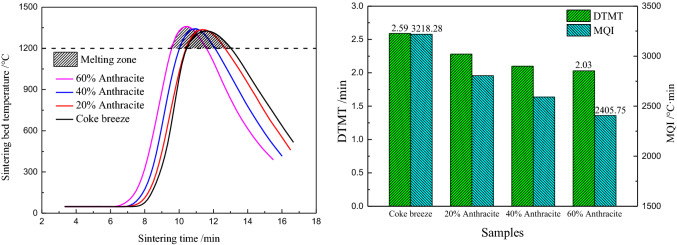
Effect of substituting anthracite for coke breeze on the thermal characteristic of the sintering bed (200 mm below the top of the sinter pot).

The effect of substituting anthracite for coke breeze on the thermal characteristic of the sintering bed was investigated at a position of 200 mm from the top of the sinter pot. The results are shown in Fig. 7. As can be seen from the temperature curves, the sinter bed temperature increased rapidly to the maximum value in about3 min, and then, it decreased relatively slowly because of the heat exchange with the suction air. Due to the different combustion characteristics of anthracite and coke breeze, the maximum temperature was reached in advance because the flame front speed increased with increasing anthracite dosage, which expedited the sintering speed. The faster combustion reactivity of anthracite resulted in a higher maximum temperature and a shorter sintering duration time, which decreases the sinter quality. As the anthracite substitution ratio increased from 0 to 60%, the melting temperature duration time decreased from 2.59 to 2.03 min, and the melt quantity index decreased from 3218.28 to 2405.75 °C·min.

**Figure 8:**
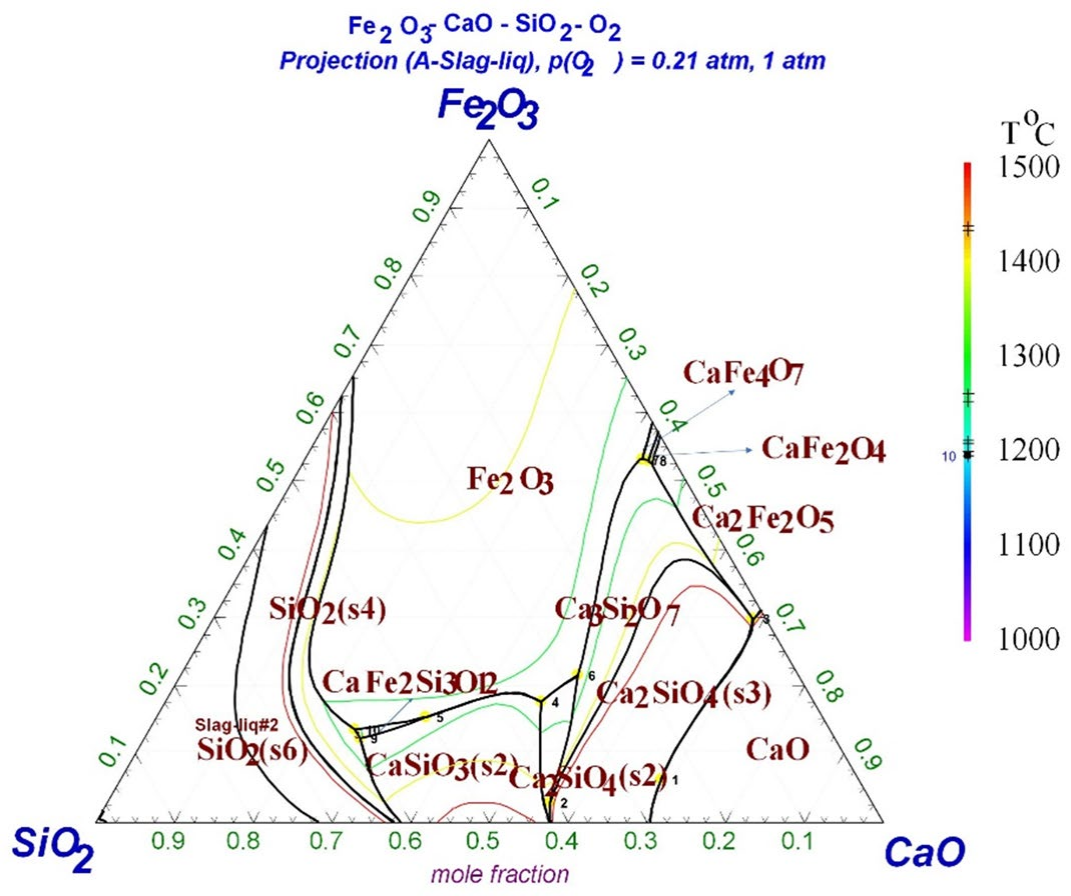
Fe_2_O_3_–SiO_2_–CaO phase diagram.

The main reactions involve the sintering process, are listed in Eqs. (5)–(10), and the characteristics and melting temperatures of the fusible compounds and the eutectic mixtures are presented in Table 4^33^. Hematite (Fe_2_O_3_), fayalite (2FeO·SiO_2_), dicalcium silicate (2CaO·SiO_2_), calcium ferrite (CaO–Fe_2_O_3_) and compound calcium ferrite (CaO–SiO_2_–Fe_2_O_3_) are the main mineral phases formed in the sintering process. The fayalite, dicalcium silicate, calcium ferrite, and compound calcium ferrite are in the liquid phase, allowing them to fill in the spaces around the hematite particles and other particles as the sintering temperature increased to about 1200 °C. This binds the particles into a firm amalgamation, especially calcium ferrite and compound calcium ferrite. The Fe_2_O_3_–CaO–SiO_2_ phase diagram was analyzed using the FactSage 7.2 software (Fig. 8). As can be seen from Fig. 8, the lowest melting point of the ternary phase is 1193 °C (point 10). The formation of compound calcium ferrite (Ca_3_Fe_2_Si_3_O_12_) induced the low melting point. The melting temperatures at points 7 and 8 are 1209 °C and 1205 °C, respectively, which is where calcium ferrites (CaFe_4_O_7_ and CaFe_2_O_4_) form. It is further demonstrated that the formation of compound calcium ferrite and calcium ferrite are quite important to the sinter quality.

**Table 4:** Main fusible compounds and eutectic mixtures in the sintering process.

System	Liquid phase characteristics	Melting temperature (°C)
${\text{Si}}{{\text{O}}_2} - {\text{FeO}}$	$2{\text{FeO}} \cdot {\text{Si}}{{\text{O}}_2}$	1205
${\text{CaO}} \cdot {\text{F}}{{\text{e}}_2}{{\text{O}}_3}$	${\text{CaO}} \cdot {\text{F}}{{\text{e}}_2}{{\text{O}}_3} \to {\text{Liquid}}\,{\text{phase}} + {\text{2Ca}} \cdot {\text{F}}{{\text{e}}_2}{{\text{O}}_3}$(Heterogenous melting compound)	1216
${\text{CaO}} \cdot {\text{F}}{{\text{e}}_2}{{\text{O}}_3}$	${\text{CaO}} \cdot {\text{F}}{{\text{e}}_2}{{\text{O}}_3} \to {\text{CaO}} \cdot 2{\text{F}}{{\text{e}}_2}{{\text{O}}_3}$(Eutectic mixture)	1205
$2{\text{CaO}} \cdot {\text{Si}}{{\text{O}}_2} - {\text{FeO}}$	$2{\text{CaO}} \cdot {\text{Si}}{{\text{O}}_2} - {\text{FeO}}$(Eutectic mixture)	1280
${\text{F}}{{\text{e}}_2}{{\text{O}}_3} - {\text{CaO}} \cdot {\text{Si}}{{\text{O}}_2}$	$2{\text{CaO}} \cdot {\text{Si}}{{\text{O}}_2} - {\text{CaO}} \cdot {\text{F}}{{\text{e}}_2}{{\text{O}}_3} - 2{\text{CaO}} \cdot {\text{F}}{{\text{e}}_2}{{\text{O}}_3}$(Eutectic mixture)	1192

Hence, the temperatures should be maintained at greater than 1200 °C, so that these reactions and the bonding process can be completed, that is, sintering mineralization can occur. When anthracite was substituted for coke breeze, the melting temperature duration time was shorter and the melt quantity index was lower, especially for an anthracite substitution ratio of 60%. Thus, the amount of sintering mineralization insufficient, leading to bad sintering indexes. Appropriate anthracite substitution ratios still meet the demand for obtaining acceptable laboratory indexes. When the anthracite substitution ratio is too high, it has a significant influence on the sintering process and results in a poor sinter quality.

5 $${\text{F}}{{\text{e}}_3}{{\text{O}}_4} + {{\text{O}}_2} \to {\text{F}}{{\text{e}}_2}{{\text{O}}_3}$$

6 $${\text{C}} + {{\text{O}}_2} \to {\text{C}}{{\text{O}}_2}$$

7 $${\text{FeO}} + {\text{Si}}{{\text{O}}_2} \to 2{\text{FeO}} \cdot {\text{Si}}{{\text{O}}_2}$$

8 $${\text{CaO}} + {\text{F}}{{\text{e}}_2}{{\text{O}}_3} \to x{\text{CaO}} \cdot y{\text{F}}{{\text{e}}_2}{{\text{O}}_3}$$

9 $${\text{CaO}} + {\text{Si}}{{\text{O}}_2} \to 2{\text{CaO}} \cdot {\text{Si}}{{\text{O}}_2}$$

10 $${\text{CaO}} + {\text{Si}}{{\text{O}}_2} + {\text{F}}{{\text{e}}_2}{{\text{O}}_3} \to x{\text{CaO}} \cdot y{\text{Si}}{{\text{O}}_2} \cdot z{\text{F}}{{\text{e}}_2}{{\text{O}}_3}$$

### Mineral composition and microstructure

**Figure 9:**
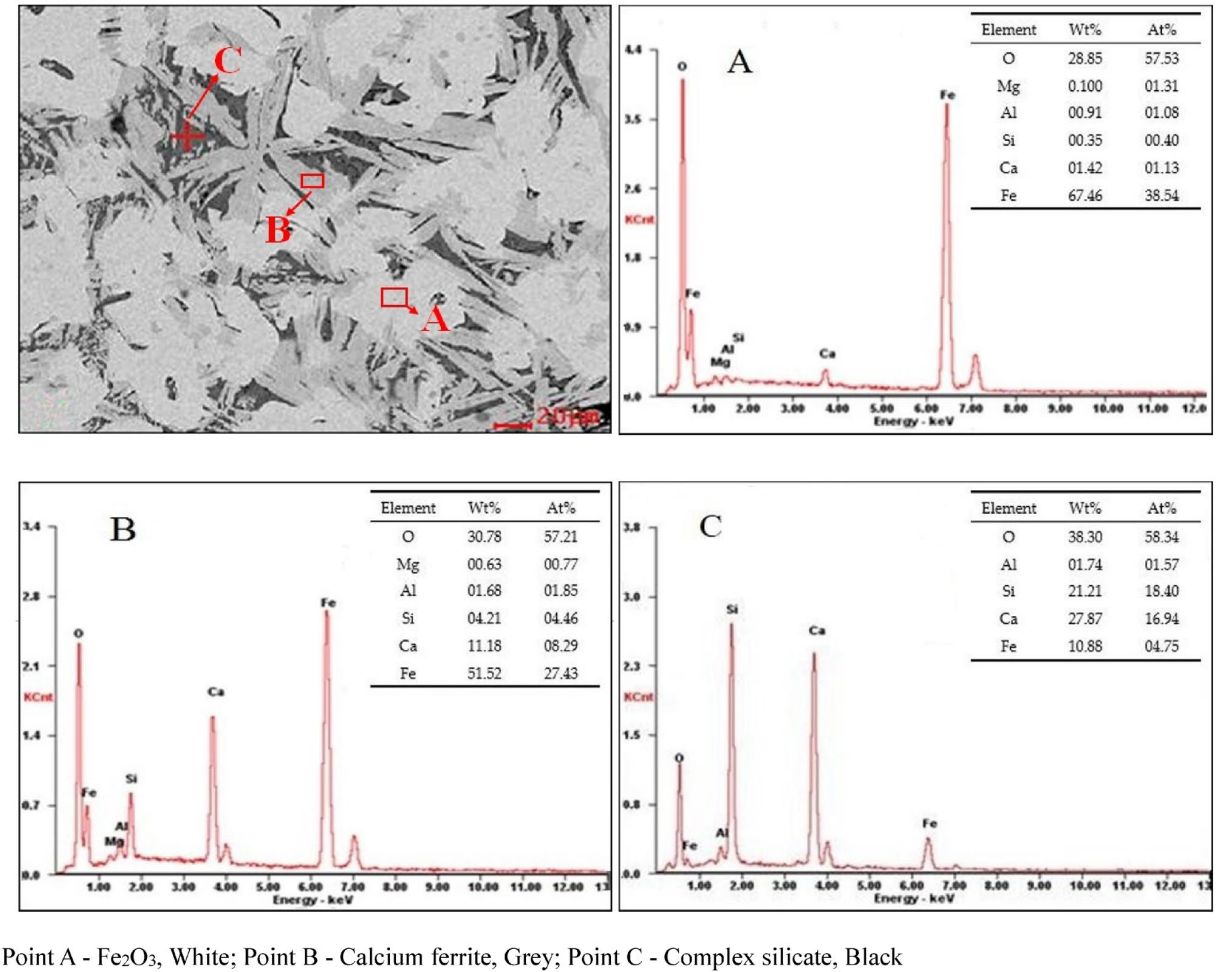
SEM images and EDS analysis results of the sinter for 40% anthracite substitution for coke breeze. Point A, Hematite (white); point B, calcium ferrite (grey); point C, complex silicate (black).

To verify the above analysis, the mineral composition of the sintered sample obtained from the 40% anthracite substitution experiment was analyzed using scanning electron microscopy (SEM) and energy dispersive X-ray spectroscopy (EDS) (Fig. 9). The three primary minerals present were hematite, calcium ferrite, and complex silicate, corresponding to points A, B, and C, respectively. The bonding phases mainly included calcium ferrite, especially SFCA, with lesser amounts of complex silicate, which contains Si, Ca, Fe, and Al.

**Figure 10:**
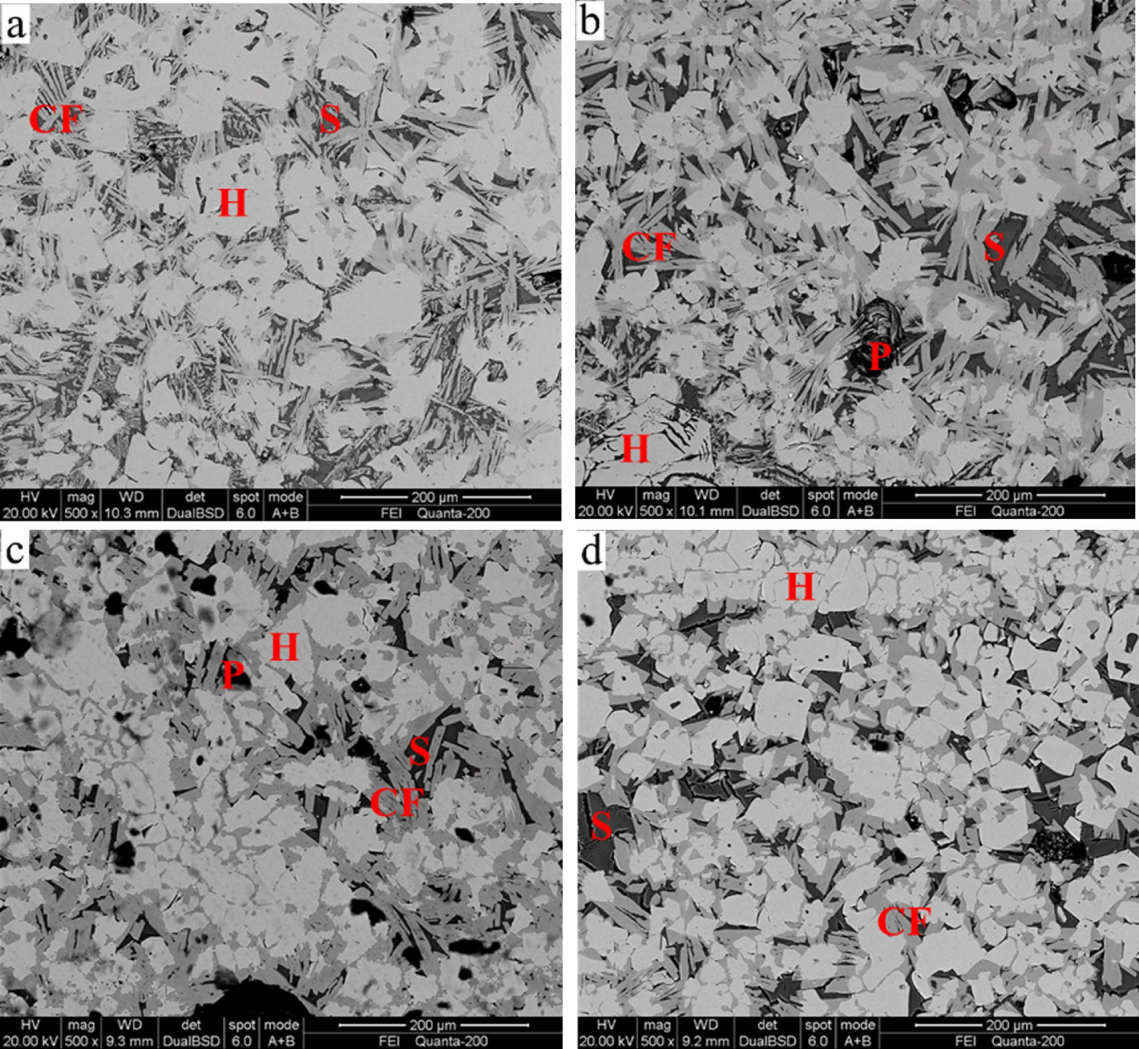
SEM images of the microstructure of the sinter for 40% anthracite substitution for coke breeze. H, Hematite (white); CF, calcium ferrite (grey); S, complex silicate (black); P, pore.

The microstructure was also a critical factor determining the properties of the sinter, which is shown in Fig. 10. The main bonding phase was acicular and stripped SFCA (Fig. 10a, b), and the granular shape is shown in Fig. 10c, d. SFCA is considered beneficial for the sinter structure (good reducibility, improves shatter index (SI) and Tumbler index (TI))^34,35^. It is closely linked to the solid particles to form an interwoven structure, which is the principal structure and has a good strength. The silicate liquid phase of the perfect crystallization closely secures the calcium ferrite to the embedded cloth shape, which is another structure with a good strength. Additionally, only a few small holes exist in the sinter. In conclusion, the fine microstructure of the sinter demonstrates that 40% anthracite substitution for coke breeze in the sintering process is feasible.

## Conclusions

The basic characteristics of anthracite and coke breeze were compared and the effects of substituting anthracite for coke breeze on the different parameters were determined through sinter pot experiments. The results indicate that anthracite can be used to partially replace coke breeze. The following is a summary of the results obtained in this study.

1. The density and porosity of anthracite are higher and lower than those of coke breeze, respectively, but they have a similar granulating performance. Both anthracite and coke breeze have similar combustion behaviors, but the combustion reactivity of anthracite is higher; hence, it requires a shorter combustion time.
2. As the anthracite proportion increases, the sintering speed and the sintering productivity increased and the tumbler index and yield decrease. The melting temperature duration time decreased from 2.59 to 2.03 min and the melt quantity index decreased from 3218.28 to 2405.75 °C·min as the anthracite ratio increased, which is disadvantageous to sintering mineralization and results in bad sintering indexes, especially for a anthracite substitution ratio of 60%.
3. The finished sinter produced by the 40% anthracite substitution experiment had a good microstructure. Calcium ferrite, Hematite, and complex silicate phase were the major mineral phases. They linked together closely and formed an interwoven structure that ensured the sintering performance.
